# Kinship Solutions for Partially Observed Multiphenotype Data

**DOI:** 10.1089/cmb.2019.0440

**Published:** 2020-09-04

**Authors:** Lloyd T. Elliott

**Affiliations:** Department of Statistics and Actuarial Science, Simon Fraser University, Burnaby, Canada.

**Keywords:** Cholesky decomposition, genome-wide association study, kinship matrix, linear mixed models, multiphenotype analysis

## Abstract

**Current work for multivariate analysis of phenotypes in genome-wide association studies often requires that genetic similarity matrices be inverted or decomposed. This can be a computational bottleneck when many phenotypes are presented, each with a different missingness pattern. A usual method in this case is to perform decompositions on subsets of the kinship matrix for each phenotype, with each subset corresponding to the set of observed samples for that phenotype. We provide a new method for decomposing these kinship matrices that can reduce the computational complexity by an order of magnitude by propagating low-rank modifications along a tree spanning the phenotypes. We demonstrate that our method provides speed improvements of around 40% under reasonable conditions.**

## 1. Introduction

Understanding the etiology and biological pathways involved in health and disease requires a multivariate analysis of phenotypic data in genome-wide association studies (GWAS). This sort of analysis is becoming more common due to increased compute power (Cox et al., 2018). Only recently have such analyses become tractable through improvements to the efficiency of linear mixed models and related models (Lippert et al., 2011; Listgarten et al., 2012, 2013; Dahl et al., 2016). Further multivariate analysis is required for the study of complex diseases such as cancer (Knox, 2010) and groups of complex phenotypes such as brain imaging phenotypes (Elliott et al., 2018), and the analysis of large consortia involving multimodal data such as the U.K. Biobank (Bycroft et al., 2018).

The use of multiphenotype (or multivariate) data often requires that a scaled version of the kinship matrix (or genetic similarity matrix, or genetic relationship matrix: Patterson et al., 2006; Balding et al., 2007) *K* be used as a covariance matrix of a multivariate distribution or matrix variate normal distribution, defined on the phenotype vector in a GWAS (Dutilleul, 1999). And so, solutions to $K^{-\frac{1}{2}}y$, in which *y* is a phenotype matrix (with a row for each sample and a column for each phenotype), must be computed to obtain maximum likelihood estimates for use in expectation maximization, variational Bayes or data whitening, or to obtain Markov chain Monte Carlo updates for Bayesian posterior simulation. Here *K* is an n×n positive-definite genetic similarity matrix, and *y* is an n×d partially observed phenotype matrix such that $y_{ij}$ is phenotype for sample *i* and phenotype *j* and *n* is the number of samples in the study and *d* is the number of phenotypes in the study. The computation of $K^{-\frac{1}{2}}y$ is also of general interest to other machine learning fields such as kernel methods (Gretton, 2003).

Phenotype matrices may be partially observed (e.g., outliers are removed leading to column-specific missingness, or experimental paradigm varies among subjects leading to blockwise missingness in phenotype categories). To compute $K^{-\frac{1}{2}}y$ for missingness in *y*, imputation may be performed (Dahl et al., 2016), but imputation can be slow and it adds additional model-specific biases into the analysis. A standard and classical method for dealing with missingness in *y* is the construction of 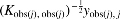 for each *j*. This is equivalent to *marginalizing out* the missing samples under the assumption of Gaussianity (i.e., it does not add additional assumptions beyond those already assumed by mixed models). Here obs(j) is the set of indices of samples observed for phenotype *j* and $A_{B,C}$ for a matrix *A* means that if *B* or *C* is a subset of N, the submatrix of *A* should be formed by selecting rows with indices in *B* or columns with indices in *C*. For ease of subscript usage, we adopt the notation $K_{j}=K_{obs\left(j\right),obs\left(j\right)}$ and $y_{j}=y_{obs\left(j\right),j}$. Computing $K_{j}^{-\frac{1}{2}}y_{j}$ for each phenotype *j* is costly and precludes scaling of multivariate GWAS analysis.

**FIG. 1.**
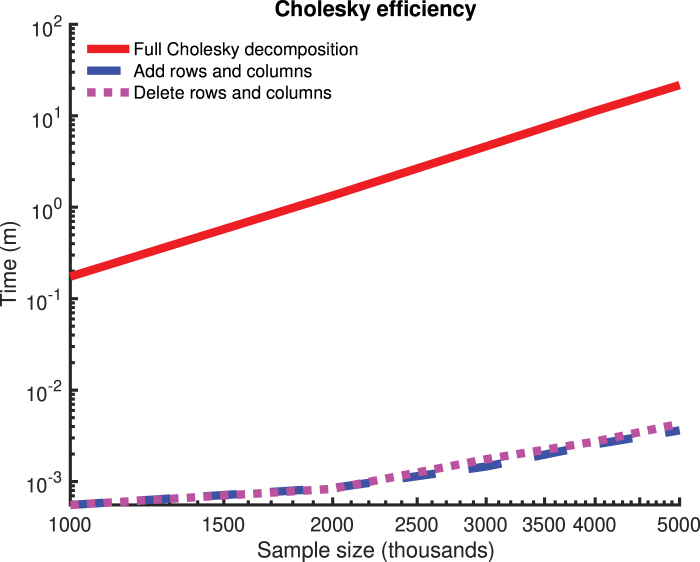
Runtimes for Cholesky decomposition and modifications for 10,000 to 50,000 samples. The log/log scale shows the asymptote and the order of magnitude relative speed of the modifications over the full Cholesky decompositions. For each condition, 15 random positive-semidefinite matrices are considered. The Cholesky decomposition is performed (mean given by red line) and then modifications are performed on a random row and column. Computations are performed using the *Intel Math Kernel Library* (MKL) implementation of the *Netlib* library.

In this article, we present an efficient algorithm for computing the Cholesky decomposition (Benoıt, 1924) of *K_j_*, for use in calculation of $K_{j}^{-\frac{1}{2}}y_{j}$. The algorithm works by computing the full $O\left(n^{3}\right)$ Cholesky decomposition $L_{j_{0}}$ for $K_{j_{0}}$ for a fixed *j*_0_, and then performing rank-1 modifications (updates and “downdates”; Benoıt, 1924) to the Cholesky decompositions and also performing other $O\left(n^{2}\right)$ operations to propagate the Cholesky decomposition of $K_{j_{0}}$ to that of $K_{j}\forall j:1\leq j\leq d,j\neq j_{0}$. The decomposition is propagated along a minimum spanning tree of a complete graph with one vertex per phenotype, and edge weights given by the number of rank-1 Cholesky modifications required for propagation of the decomposition along that edge. In Figure 1, a comparison is provided for the runtime and asymptotics of the full Cholesky decomposition against the Cholesky modifications for a range of sample sizes, indicating the efficiency of these modifications. In this figure, genetic similarity matrices are simulated by drawing from a Wishart distribution with means given by the identity matrix and with 10,000 degrees of freedom (simulating a study typed at 10,000 markers). We refer to our algorithm as *kgen* (for *k*inship *gen*eration) and we implement our algorithm in an open source software package called the *kgen* software. A manual for this software is provided in Appendix C of the Supplementary Material.

The asymptotic complexity of performing Cholesky decompositions in a naive way for all *K_j_* is $O\left(dn^{3}\right)$, (assuming the missingness patterns of each phenotype are not identical). In contrast, the worst-case asymptotic complexity of the *kgen* algorithm is as follows:

(1) $$O\left(n^{3}+rdn^{2}\right).$$

Here *r* is defined as $\max_{1\leq j_{1}<j_{2}\leq d}\left\{\#\left(obs\left(j_{1}\right)\setminus obs\left(j_{2}\right)\right)+\#\left(obs\left(j_{2}\right)\setminus obs\left(j_{1}\right)\right)\right\}$ (this is the maximum number of rank-1 Cholesky modifications required to propagate a Cholesky decomposition between two phenotypes) and #A denotes the size of a set *A* and B∖C for sets *B* and *C* denotes the set difference between *B* and *C*.

### 1.1. Related work

To reduce the computational complexity of genetic similarity matrix operations, several research programs have been conducted to store and manipulate sparse representations of the genetic similarity matrix (Shor et al., 2019). In these representations, researchers set a threshold and then zero out elements of the genetic similarity matrix with absolute value less than this threshold. The computational gains of such an approach may be large, but in theory, such an approach could lead to loss of power. Furthermore, this approach would be less suitable to data originating from pedigrees or small isolated populations. To our knowledge, our work is the first to leverage Cholesky low-rank modifications for improving efficiency of genetic similarity matrix-based inference.

## 2. Methods

In this section, we describe the genetic similarity matrices for which the *kgen* algorithm is appropriate and then provide the details of the *kgen* algorithm.

### 2.1. Genetic similarity matrices

The definition of the kinship matrix we use is that of a genetic similarity matrix centered at population-level minor allele frequencies. This definition is based on Patterson et al. (2006) but note that it involves population-level normalization instead of sample-level normalization. Let $G_{i\ell }$ be the genotype of the *i*-th subject at the ℓ-th marker (suppose that there are n markers in total), and let $\rho_{\ell }$ be the minor allele frequency of the ℓ-th marker with respect to the population from which the samples are drawn (we assume 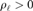). Then, the genetic similarity matrix is an n×n positive semidefinite matrix such that:

(2) $$K_{i_{1},i_{2}}=\frac{1}{m}\sum_{\ell =1}^{m}\frac{\left(G_{i_{1},\ell }-2\rho_{\ell }\right)\left(G_{i_{2},\ell }-2\rho_{\ell }\right)}{2\rho_{\ell }\left(1-\rho_{\ell }\right)}.$$

The Cholesky decomposition of an n×n positive definite matrix *K* is the unique upper triangular matrix *L* such that $LL^{T}=K$ and so $L^{-1}y$ is a solution to $K^{-\frac{1}{2}}y$. Efficient algorithms exist for computing *L* (Anderson et al., 1999), and although the computation of *L* has asymptotic complexity $O\left(n^{3}\right)$, it is often much faster than a matrix inversion performed on a matrix of the same size. Due to the upper triangular nature of *L*, $L^{-1}$ and $L^{-1}y$ may be computed with asymptotic complexity $O\left(n^{2}\right)$. Given a Cholesky decomposition *L* of *K*, for any vector *v* of length *n*, the Cholesky decomposition *L* may be updated to form the Cholesky decomposition of $K+vv^{T}$ (i.e., the sum of *K* and the rank-1 vector $vv^{T}$) or downdated to form the Cholesky decomposition of $K-vv^{T}$. These update and downdate operations have asymptotic complexity $O\left(n^{2}\right)$. For more detail on Cholesky decompositions and their modifications, we refer to Seeger (2008), Osborne et al. (2010), and Benoıt (1924).

### 2.2. The *kgen* algorithm

A meditation on these asymptotes suggests the *kgen* algorithm. The Cholesky modifications can be designed to add and remove rows and columns of *K* (Osborne et al., 2010), and so, the computation of $K_{1}^{-\frac{1}{2}}y_{1},\ldots ,K_{d}^{-\frac{1}{2}}y_{d}$ can be done by performing only one $O\left(n^{3}\right)$ full Cholesky decomposition (instead of *d* such operations) and then propagating it to the rest of the decompositions, provided that the number of rows and columns to be added and removed to propagate the Cholesky decompositions is small with respect to *n* yielding Eq. (1).

Procedures to arrange Cholesky modifications in a way that adds and removes rows and columns of *K* are provided in Osborne et al. (2010). We refer to these operations as *delete* and *insert*. These operations are described formally as follows. Let *v* be an n×1 vector and let *L* be the Cholesky decomposition of the n×n positive definite matrix

(3) $$K^{+}=\left(\begin{array}{ccc} K_{\left\{1,\ldots ,i-1\right\},\left\{1,\ldots ,i-1\right\}} & v_{\left\{1,\ldots ,i-1\right\},1} & K_{\left\{1,\ldots ,i-1\right\},\left\{i+1,\ldots ,n\right\}}\\ v_{\left\{1,\ldots ,i-1\right\},1}^{T} & v_{i1} & v_{\left\{i+1,\ldots ,n\right\},1}^{T}\\ K_{\left\{i+1,\ldots ,n\right\},\left\{1,\ldots ,i-1\right\}} & v_{\left\{i+1,\ldots ,n\right\},1} & K_{\left\{i+1,\ldots ,n\right\},\left\{i+1,\ldots ,n\right\}} \end{array}\right).$$

Then, $L^{-}=delete\left(K^{+},i\right)$ is the Cholesky decomposition of

(4) $$K^{-}=\left(\begin{array}{cc} K_{\left\{1,\ldots ,i-1\right\},\left\{1,\ldots ,i-1\right\}} & K_{\left\{1,\ldots ,i-1\right\},\left\{i+1,\ldots ,n\right\}}\\ K_{\left\{i+1,\ldots ,n\right\},\left\{1,\ldots ,i-1\right\}} & K_{\left\{i+1,\ldots ,n\right\},\left\{i+1,\ldots ,n\right\}} \end{array}\right).$$

Conversely, let *L* be the Cholesky decomposition of the matrix $K^{-}$ given in Equation (4), then $L^{+}=insert\left(K^{-},i,v\right)$ is the Cholesky decomposition of the matrix $K^{+}$ given in Equation (3).

These *insert* and *delete* operations can be performed in $O\left(n^{2}\right)$ time. Descriptions of these operations are provided in Algorithms S1 and S2 of the Supplementary Material. For our *delete* operation, we use the procedure from Osborne et al. (2010). For our *insert* operation, we use a procedure slightly different from Osborne et al. (2010) and provide a proof of our procedure in Appendix A of the Supplementary Material.

We now describe the *kgen* algorithm in detail. The *kgen* algorithm is listed in Algorithm 1 in this article. This algorithm assumes that an n×n positive definite genetic similarity matrix *K* is provided as in Eq. (2), and that an n×d phenotype matrix *Y* is provided, with missing entries indicated. The phenotype matrix is used to find the sets of missing entries obs(j):1≤j≤d, and the particular values of the phenotype matrix are not used. Instead, Cholesky decompositions *L_j_* of $K_{j}=K_{obs\left(j\right),obs\left(j\right)}$ are returned, providing fast access to $L_{j}^{-1}y_{j}$.

The *kgen* algorithm works by first finding a phenotype *j*_0_ such that the number of missing entries for the *j*_0_-th column of *Y* is less than or equal to the number of missing phenotypes in any other column. And then, we find a tree spanning all phenotypes. The tree is chosen such that the sum of the number of samples that must be added or removed for each edge of the tree (the sum of the weights of the edges) is minimized. For an edge from phenotypes *j*_1_ to *j*_2_, a sample must be added if it is in $obs\left(j_{2}\right)$ but not in $obs\left(j_{1}\right)$, and a sample must be removed if it is in $obs\left(j_{1}\right)$ but not in $obs\left(j_{2}\right)$. Note that this relation about the number of samples to be added or removed is reflexive and so the weighted complete graph with vertices given by the columns of the phenotype matrix is an undirected graph. The vertex *j*_0_ is identified as the root of this minimum spanning tree. In our implementation of this algorithm, we use Kruskal's algorithm to find the minimum spanning tree (Kruskal, 1956).

After this minimum spanning tree is created, a breadth-first enumeration of the edges of this tree is constructed, such that the first edge includes the root of the tree. This enumeration must be breadth-first, because propagation of Cholesky decompositions along an edge may involve hard-drive reads and writes. The finished decompositions may have to be written and read to disk, as RAM (random access memory) provisions on supercomputers often cannot store the Cholesky decompositions of >1,000 phenotypes with 20,000 samples. So, software implementing the *kgen* algorithm will read the decomposition from the “source” vertex unless that decomposition has been recently read. Ensuring that the decomposition for the “source” vertex has most often been recently read is equivalent to providing the path through the spanning tree in a breadth-first way.

**Table:** 

**Algorithm 1** The *kgen* algorithm
1: **Inputs:** a) An *n* × *n* positive definite matrix *K*; b) An *n* × *d* phenotype matrix *Y*.
2: **Outputs:** A list of Cholesky decompositions *L_j_* for 1 ≤ *j* ≤ *d* wherein *L_j_* is the #obs(*j*) × #obs(*j*) Cholesky decomposition of the positive definite matrix *K_j_*.
3: Let *G* be the weighted undirected complete graph on *d* vertices such that the weight of the edge between vertices *j*_1_ and *j*_2_ is #(obs(*j*_1_) \obs(*j*_2_)) + #(obs(*j*_2_) \obs(*j*_1_)).
4: Let *T* be a minimum spanning tree of *G*.
5: Let *j*_0_ be a vertex such that #obs(*j*_0_) ≤ #obs(*j*) ∀ 1 ≤ *j* ≤ *d*.
6: Let *E*_1_,…, *E_d_*_−1_ be a breadth-first enumeration of all of the edges of *T* along with an ordering of the vertices of each edge (so the two vertices defining the edge *E_i_* are given in order by *E_i_*_1_ and *E_i_*_2_), such that the vertex *E_i_*_1_ always appears among the set {*j*_0_, *E*_12_,…, *E_i_*_−1,2_} and such that *E*_11_ = *j*_0_.
7: *L_j_*_0_ ← chol(*K_j_*_0_)
8: **for***j* = 1 … *d* – 1 **do**
9: *e*_1_ ← *L_Ej_*_1_
10: *e*_2_ ← *L_Ej_*_2_
11: *L*′ ← *L_e_*_1_
12: *S* ← obs(*e*_1_)
13: *k* ← 1
14: **for***i* = 1 … *n***do**
15: **if***i*∈obs(*e*_1_) and *i*∈obs(*e*_2_) **then**
16: *k* ← *k* + 1
17: **else if***i*∈obs(*e*_1_) and *i* ∉ obs(*e*_2_) **then**
18: *S* ← *S* \{*i*}
19: *L*′ ← *delete*(*L*′, *k*)
20: **else if***i* ∉ obs(*e*_1_) and *i*∈obs(*e*_2_) **then**
21: *S* ← *S* ∪{*i*}
22: *L*′ ← *insert*(*L*′, *K_S,k_*, *k*)
23: *k* ← *k* + 1
24: **assert***S* = obs(*e*_2_)
25: *L*_e2_ ← L′
26 **return***L*_1,…,_*L_d_*

After the enumeration is created, the *kgen* algorithm computes the Cholesky decomposition of $K_{j_{0}}$ and then for each edge $\left(e_{1},e_{2}\right)$ in the enumeration, it modifies the Cholesky decomposition of $K_{e_{1}}$ by inserting samples that are in $obs\left(e_{2}\right)\setminus obs\left(e_{1}\right)$ and deleting samples that are in $obs\left(e_{1}\right)\setminus obs\left(e_{2}\right)$. And then it proceeds to the next edge in the path, and repeats this procedure until the d−1 edges in the minimum spanning tree on the phenotypes are exhausted.

### 2.3. A worked example of the *kgen* algorithm

**FIG. 2.**
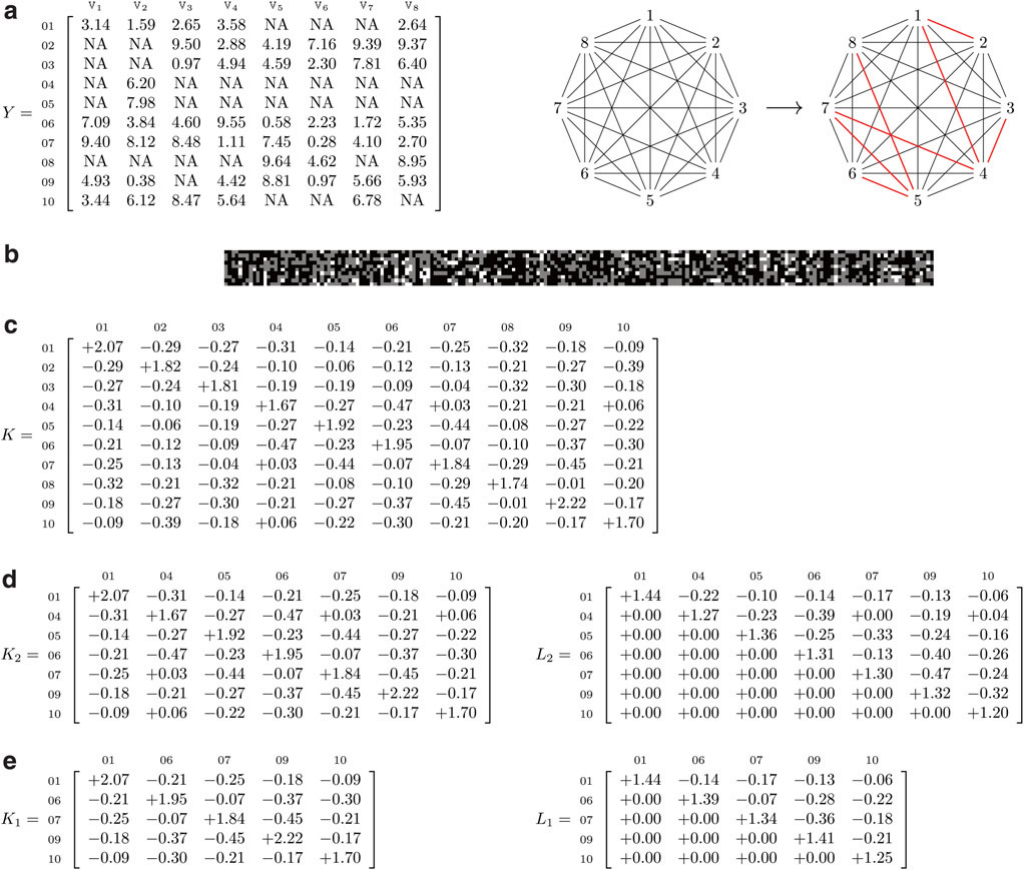
A worked example for the *kgen* algorithm. **(a)** Left: The exact values of the phenotypes *Y*. Missing data are denoted by the string NA. Middle: A complete undirected weighted graph on the 8 phenotypes. Weights are omitted, but can be inferred from *Y*: (e.g., the weight between phenotypes one and two is two, as two samples have to be inserted to move from phenotype one to phenotype two). Right: The minimum spanning tree over the phenotypes. **(b)** The simulated genotypes (*x*-scale indicates marker index and *y*-scale indicates sample index, and colors are white for homozygous minor, gray for heterozygous, and black for homozygous major). **(c)** The kinship matrix implied by the genotypes (to two decimals of precision). **(d, e)** The kinship matrix restricted to the observed subjects for phenotype two or one *(resp.)* and the corresponding Cholesky decompositions.

In Figure 2, we provide a worked example of the *kgen* algorithm involving 10 samples and 8 phenotypes. In this example, the root of the minimum spanning tree is phenotype two and a breadth-first enumeration of the edges of the minimum spanning tree (such that the root is given by the first edge) is (2,1),(1,4),(4,3),(4,7)…. The Cholesky decomposition in Fig. 2e right can be formed by performing rank-1 modifications after computing the Cholesky decomposition in Fig. 2d.

## 3. Experiments

We now consider two experiments on simulated data and compare the speed and accuracy of the *kgen* algorithm to that of a naive algorithm in which the full Cholesky decomposition is computed for each phenotype. In the first experiment, we vary the number of samples and the missingness rate of the phenotype measurements, and assume that the data are missing-at-random, and assume a fixed number of 100 phenotypes per condition. In the second experiment, we examine a blockwise missingness pattern.

These experiments (and the results are displayed in Fig. 1) were conducted on Intel Xeon E5-2683 CPUs, and the numerical matrix operations were performed using Intel's *MKL* (math kernel library) implementation of the *Netlib* library (Anderson et al., 1999). The machine epsilon on this CPU was 2.2e−308.

### 3.1. Experiment 1

**FIG. 3.**
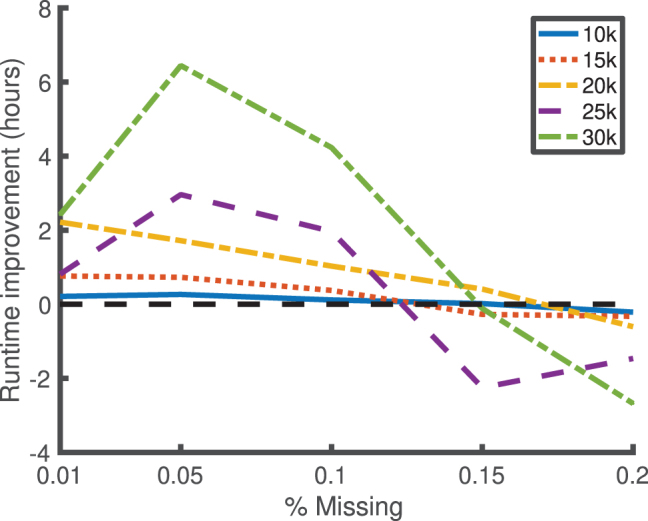
Improvement in runtime for the *kgen* software over naive Cholesky decompositions for low values of missingness (*x*-scale) for 100 phenotypes, and varying numbers of samples (indicated by legend). The *y*-scale indicates the median runtime for the *kgen* software minus the runtime of naive Cholesky decompositions, over five replicates per condition. The *kgen* software is better for all values of missingness ≤0.1%.

We consider missingness rates of 0.01%, 0.05%, 0.1%, 0.15%, and 0.2%, and a missing-at-random missingness pattern over 100 phenotypes. We consider *n* = 10,000, 15,000, 20,000, 25,000, and 30,000 samples. For each condition, we consider five independent replicates, and we sample the kinship matrix from the same Wishart distribution that was used in Figure 1 (i.e., with a mean given by the identity matrix and with 10,000 degrees of freedom). The difference in the runtime between *kgen* and the naive method (in which a full Cholesky decomposition is done for each phenotype), averaged over the five independent replicates for each condition, is displayed in Figure 3. The maximum entrywise absolute difference between the two methods over all phenotypes and conditions was 1.1768*e* − 14 (in the units of the Cholesky decomposition space), indicating close alignment and low numerical imprecision.

A histogram of all of the elementwise absolute differences is displayed in Supplementary Figure S1 in the Supplementary Material. The runtime of each replicate (for both the *kgen* algorithm and the naive method) and the maximum entrywise absolute difference between the Cholesky decompositions of each method are shown in Supplementary Table S1 of Appendix B in the Supplementary Material.

### 3.2. Experiment 2

**FIG. 4.**
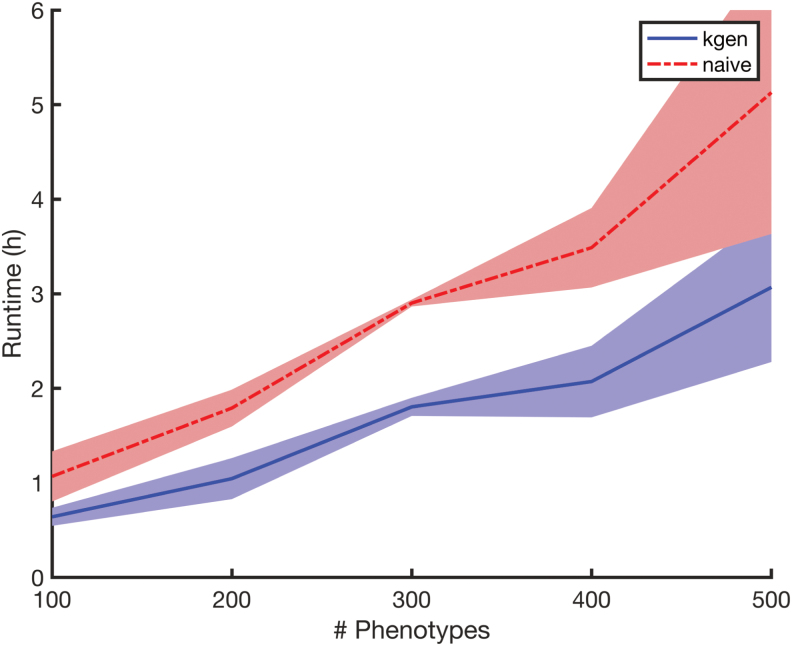
Runtimes for Experiment 2 versus number of phenotypes, displaying some linearity in *d* (varied in the set {100,200,300,400,500}). Lines show means over five independent replicates. Shaded region indicates standard deviations. The *kgen* algorithm improves runtimes by ∼40% in all conditions.

In our second experiment, we consider a blockwise missingness pattern and vary the number of phenotypes. We fix the number of samples at n=15,000 and we consider a missing-at-random rate of 0.1% and also a blockwise missingness pattern in which each block of 50 consecutive phenotypes all have 10% of the samples masked (the same 10% of samples are masked for all 50 phenotypes in the block). The genetic similarity matrix is taken to be the same Wishart distribution that was used in Experiment 1. This situation is similar to a massively multiphenotyped version of the Wellcome Trust Case/Control Consortium (2007). The number of phenotypes is varied in the set {100,200,300,400,500}. The runtime of *kgen* and the naive method for Experiment 2 are shown in Figure 4.

Improvements shown in Figure 3 are only generally realized for <0.1% missingness. And so for Experiment 2 (and our released *kgen* software), we institute a new rule in which if >100 samples must be added or removed to propagate along an edge between one phenotype and another in the minimum spanning tree, instead a full Cholesky decomposition is performed on the other phenotype. This “short circuits” the *kgen* algorithm and allows superb performance even in cases for which the overhead of the low-rank modifications in the *kgen* algorithm could swamp the gains (the choice of 100 depends on the overhead and could be reduced in the future, to respect new and more powerful hardware).

## 4. Results

In Figure 3, we see a between 1- and 6-hour improvement for sample sizes greater than 15,000 and missingness rates <0.1%, and for 100 phenotypes. In theory, and in Figure 4, we see an indication that these improvements are linear in the number of phenotypes. And so a study with 10,000 phenotypes under the right conditions may benefit from a 25-day reduction in compute on our hardware through the *kgen* algorithm.

Since Figure 3 indicates the difference in runtimes between *kgen* and the naive method, we also provide means and standard deviations for the runtime for both methods in Table 1. This table indicates that the *kgen* algorithm provides between 0% and 40% improvement in runtime for missingness at random rates of less than 0.1%. Figure 4 indicates around a 40% improvement for situations similar to The Wellcome Trust Case/Control Consortium (2007).

**Table 1.** Detailed Results for Experiment 1

*n*	Rate	Mean (*kgen*)	Std (*kgen*)	Mean (*naive*)	Std (*naive*)
10,000	01	283	81.781	1044	175.907
10,000	05	492	233.195	1457	254.245
10,000	10	983	486.558	1364	276.035
10,000	15	1352	610.731	1282	283.564
10,000	20	2572	940.290	1896	678.998
15,000	01	502	81.288	3276	274.027
15,000	05	1052	134.875	3875	276.780
15,000	10	3260	1121.938	4314	446.635
15,000	15	5461	1940.624	4472	711.593
15,000	20	5345	1162.868	3910	319.418
20,000	01	1270	413.137	9029	1105.049
20,000	05	2521	321.002	9080	855.493
20,000	10	6650	1891.464	9654	1267.275
20,000	15	9943	3244.624	10,422	1403.009
20,000	20	13,540	4436.192	10,156	2372.845
25,000	01	567	231.747	4368	2256.119
25,000	05	8188	3164.044	20,674	3322.788
25,000	10	11,276	810.770	18,165	1055.435
25,000	15	24,398	7299.440	18,742	1989.225
25,000	20	24,460	1885.233	20,427	6236.077
30,000	01	1266	960.024	10,743	8736.785
30,000	05	13,200	5850.212	34,280	3823.599
30,000	10	24,727	6558.746	32,808	4861.380
30,000	15	31,193	5695.082	31,005	4110.901
30,000	20	40,397	5455.380	29,346	5139.770

The “*n*” column indicates number of samples, “rate” column indicates the missingness rate (in basis points), “mean (*kgen*)” and “std (*kgen*),” “mean (*naive*)” and “std (*naive*)” column indicates means and standard deviations over five independent replicates for *kgen* and the naive method (in seconds) *resp*.

## 5. Conclusion

Multivariate GWAS are limited by computational resources. We have provided a new method to create and manipulate the genetic similarity matrices required for linear mixed models for multivariate GWAS. On our hardware, our method provides an improvement of around 40% under reasonable simulation settings. Software implementing our methods are released under an open source license.

## Supplementary Material

Supplemental data

## References

[B1] Anderson E., Bai Z., Bischof C., et al. 1999 LAPACK Users' Guide, 3rd ed. Society for Industrial and Applied Mathematics. San Diego, CA

[B2] Balding D J., Bishop M., and Cannings C. 2007 Handbook of Statistical Genetics. Wiley-Interscience

[B3] Benoıt E. 1924 Note sur une méthode de résolution des équations normales provenant de l'application de la méthode des moindres carrésa un systeme d'équations linéaires en nombre inférieura celui des inconnues. (Procédé du Commandant Cholesky). Bulletin Godsique 2, 67–77

[B4] Bycroft C., Freeman C., Petkova D., et al. 2018 The UK Biobank resource with deep phenotyping and genomic data. Nature 562 10.1038/s41586-018-0579-z PMC6786975 30305743

[B5] Cox D.R., Kartsonaki C., and Keogh R.H. 2018 Big data: Some statistical issues. Stat. Probab. Lett. 136, 111–115 29899584 10.1016/j.spl.2018.02.015 PMC5992743

[B6] Dahl A., Iotchkova V., Baud A., et al. 2016 A multiple-phenotype imputation method for genetic studies. Nat. Genet. 48, 466–472 26901065 10.1038/ng.3513 PMC4817234

[B7] Dutilleul P. 1999 The MLE algorithm for the matrix normal distribution. J. Stat. Comput. Simul. 64, 105–123

[B8] Elliott L.T., Sharp K., Alfaro-Almagro F., et al. 2018 Genome-wide association studies of brain imaging phenotypes in UK Biobank. Nature 562, 210–216 30305740 10.1038/s41586-018-0571-7 PMC6786974

[B9] Gretton A. 2003 Kernel Methods for Classification and Signal Separation. (Doctoral Thesis). University of Cambridge, Cambridge, UK

[B10] Knox S.S. 2010 From ‘omics’ to complex disease: A systems biology approach to gene-environment interactions in cancer. Cancer Cell Int. 10, 11 20420667 10.1186/1475-2867-10-11 PMC2876152

[B11] Kruskal J.B. 1956 On the shortest spanning subtree of a graph and the traveling salesman problem. Proc. Am. Math. Soc. 7, 48–50

[B12] Lippert C., Listgarten J., Liu Y., et al. 2011 FaST linear mixed models for genome-wide association studies. Nat. Methods 8, 833–835 21892150 10.1038/nmeth.1681

[B13] Listgarten J., Lippert C., and Heckerman D. 2013 FaST-LMM-Select for addressing confounding from spatial structure and rare variants. Nat. Genet. 45, 470–471 23619783 10.1038/ng.2620

[B14] Listgarten J., Lippert C., Kadie C.M., et al. 2012 Improved linear mixed models for genome-wide association studies. Nat. Methods 9, 525–526 22669648 10.1038/nmeth.2037 PMC3597090

[B15] Osborne M.A., Rogers A., Roberts S.J., et al. 2010 Bayesian Gaussian process models for multi-sensor time-serie prediction. In Inference and Learning in Dynamic Models. Barber, D., et al. (Eds): Cambridge University Press

[B16] Patterson N., Price A.L., and Reich D. 2006 Population structure and eigenanalysis. PLoS Genet. 2, e190 17194218 10.1371/journal.pgen.0020190 PMC1713260

[B17] Seeger M. 2008 Low Rank Updates for the Cholesky Decomposition (Technical Report). University of California at Berkeley

[B18] Shor T., Kalka I., Geiger D., et al. 2019 Estimating variance components in population scale family trees. PLoS Genet. 15, e1008124 31071088 10.1371/journal.pgen.1008124 PMC6529016

[B19] The Wellcome Trust Case Control Consortium. 2007 Genome-wide association study of 14,000 cases of seven common diseases and 3,000 shared controls. Nature 447, 661–678 17554300 10.1038/nature05911 PMC2719288

