# Supplementary material for: Kinship Solutions for Partially Observed Multiphenotype Data
Source: J Comput Biol. 2020 Sep 4;27(9):1461–70. doi: 10.1089/cmb.2019.0440 (PMC7482112; doi:10.1089/cmb.2019.0440)
Supplement: Supplemental data [file Supp_Data.zip › kgen-master/supmat.pdf]

Kinship solutions for partially observed multi-phenotype data

Supplementary Material

Lloyd T. Elliott*

Department of Statistics and Actuarial Science
Simon Fraser University, Canada

*8888 University Drive, Burnaby, B.C. V5A 1S6.

Appendix A: Algorithm for *insert* and *delete* operations

In this Appendix, we provide algorithms for the *insert* and *delete* operations on Cholesky decompositions. These operations modify a Cholesky decomposition L^+ or L^- of a positive definite *(p.d.)* matrix A to form the Cholesky decomposition L' of a new matrix A' in which a row and column are added or remove *resp.* from A.

We refer to the two auxiliary operations indicating the transformation $A \mapsto A'$ through adding or removing rows and columns by *augment* and *diminish resp.* and we specify them here. The augmentation operation is denoted $A' = \mathit{augment}(A, v, i)$. Here A is an $n-1 \times n-1$ matrix and v is an $n \times 1$ vector. This operation is given by the following display.

$$A' = \begin{pmatrix} A_{11} & \cdots & A_{1,i-1} & v_1 & A_{1,i} & \cdots & A_{1,n-1} \\ \vdots & \ddots & \vdots & \vdots & \vdots & \ddots & \vdots \\ A_{i-1,1} & \cdots & A_{i-1,i-1} & v_{i-1} & A_{i-1,i} & \cdots & A_{i-1,n-1} \\ v_1 & \cdots & v_{i-1} & v_i & v_{i+1} & \cdots & v_n \\ A_{i,1} & \cdots & A_{i,i-1} & v_{i+1} & A_{i,i} & \cdots & A_{i,n-1} \\ \vdots & \ddots & \vdots & \vdots & \vdots & \ddots & \vdots \\ A_{n-1,1} & \cdots & A_{n-1,i-1} & v_n & A_{n-1,i} & \cdots & A_{n-1,n-1} \end{pmatrix} \tag{1}$$

Similarly, the diminishing operation removes the i-th row and column from an $n \times n$ matrix and is denoted $A' = \mathit{diminish}(A, i)$ and is given by the following display.

$$A' = \begin{pmatrix} A_{11} & \cdots & A_{1,i-1} & A_{1,i+1} & \cdots & A_{1,n} \\ \vdots & \ddots & \vdots & \vdots & \ddots & \vdots \\ A_{i-1,1} & \cdots & A_{i-1,i-1} & A_{i-1,i+1} & \cdots & A_{i-1,n} \\ A_{i+1,1} & \cdots & A_{i+1,i-1} & A_{i+1,i+1} & \cdots & A_{i+1,n} \\ \vdots & \ddots & \vdots & \vdots & \ddots & \vdots \\ A_{n1} & \cdots & A_{n,i-1} & A_{n,i+1} & \cdots & A_{n,n} \end{pmatrix} \tag{2}$$

The specification for the *insert* and *delete* operations (referenced in Algorithm 1 of the main text) are now provided in Algorithms S1 and S2.

Algorithm S1 *insert*

Inputs: a) A Cholesky decomposition L^{-} of an $n-1 \times n-1$ *p.d.* matrix A; b) An integer $1 \leq i \leq n-1$ indicating the index of the row and column that is to be inserted; c) An $n \times 1$ vector v specifying the values of the inserted row and column.
Outputs: Cholesky decomposition L' of $A' = augment(A, v, i)$.
$L' \leftarrow augment(L^{-}, 0_{n\times 1}, i)$
$v' \leftarrow v$
$v'_i \leftarrow v_i/2$
$v' \leftarrow v'/\|v'\|$
$u \leftarrow 0_{n\times 1}$
$u_i \leftarrow 1$
$x \leftarrow (\|v'\|/2)^{\frac{1}{2}}(u + v')$
$y \leftarrow (\|v'\|/2)^{\frac{1}{2}}(u - v')$
$L' \leftarrow \text{udate}(L', x)$
$L' \leftarrow \text{ddate}(L', y)$
return L'

Algorithm S2 *delete*

Inputs: a) A Cholesky decomposition L^{+} of an $n \times n$ *p.d.* matrix A; b) An integer $1 \leq i \leq n$ indicating the index of the row and column that is to be deleted.
Outputs: Cholesky decomposition L' of $A' = diminish(A, i)$.
$L' \leftarrow L^{+}_{i+1:n,i+1:n}$
$v \leftarrow \left(L^{+}_{i,i+1:n}\right)^T$
$L' \leftarrow \text{udate}(L', v)$
$L \leftarrow L^{+}$
$L_{i+1:n,i+1:n} \leftarrow L'$
$L' \leftarrow diminish(L, i)$
return L'

Here $udate(L, x)$ and $ddate(L, y)$ refer to the rank-1 Cholesky update and downdate operations (Benoıt, 1924) and $0_{n\times 1}$ denotes the zero vector with n rows and A^T denotes the transpose of the matrix A. We use the *udate* and *ddate* implementation from the *qrupdate* library (Hájek, 2012) in our implementation of these algorithms.

Algorithm S2 is based on Osborne, Rogers, Roberts, Ramchurn, and Jennings (2010) and the proof of Algorithm S2 is contained in that reference. The proof of Algorithm S1 is as follows. With the notation established in the

preconditions of Algorithm S1, let $A_0 = \mathit{augment}(A, 0_{n+1\times 1}, i)$. Let

$$v' = \frac{(v_1, \ldots, v_{i-1}, v_i/2, v_{i+1}, \ldots, v_n)^T}{\|(v_1, \ldots, v_{i-1}, v_i/2, v_{i+1}, \ldots, v_n)^T\|}, \qquad u = \Bigg(\underbrace{0, \ldots, 0}_{i-1 \text{ times}}, 1, \underbrace{0, \ldots, 0}_{n-i \text{ times}}\Bigg)^T, \quad (3)$$

$$x = \sqrt{\frac{\|v'\|}{2}}(u + v'), \qquad y = \sqrt{\frac{\|v'\|}{2}}(u - v'). \quad (4)$$

Then, $\mathit{augment}(A, v, i) = A_0 + xx^T - yy^T$. The matrix $\mathit{augment}(L^-, 0_{n\times 1}, i)$ is the Cholesky decomposition of A_0, and so ddate(udate$(L', x), y)$ is the Cholesky decomposition of $\mathit{augment}(A, v, i)$.

Appendix B: Extended data for Experiment 1

The Table S1 provides the raw results for Experiment 1 in the main text. The first column `C` provides the condition (1 = *naïve* algorithm and 2 = *kgen* algorithm), and the second column `P` provides the number of samples, and the third column `R` provides the amount of missingness at random (in basis points), and the fourth column `T` provides the trial number (between 1 and 5, inclusive), and the fifth column `Y` provides the runtime (in seconds), and the sixth column `D` provides the maximum absolute entrywise difference between the two methods over all trials and phenotypes for each condition (in the units of the Cholesky decomposition space). The number of phenotypes used in each replicate is 100. The string `Inf` indicates the value ∞.

C	P	R	T	Y	D
1	10000	01	1	1189	Inf
1	10000	01	2	831	15.65
1	10000	01	3	904	15.65
1	10000	01	4	1238	15.26
1	10000	01	5	1056	Inf
1	10000	05	1	1311	15.35
1	10000	05	2	1375	15.18
1	10000	05	3	1800	15.11
1	10000	05	4	1630	15.18
1	10000	05	5	1169	15.05

C	P	R	T	Y	D
1	10000	10	1	1799	14.95
1	10000	10	2	1133	14.88
1	10000	10	3	1168	14.95
1	10000	10	4	1470	14.75
1	10000	10	5	1252	15.18
1	10000	15	1	1142	14.95
1	10000	15	2	1789	14.88
1	10000	15	3	1173	14.57
1	10000	15	4	1141	14.45
1	10000	15	5	1167	14.40

C	P	R	T	Y	D
1	10000	20	1	1808	14.75
1	10000	20	2	1302	14.65
1	10000	20	3	1730	14.57
1	10000	20	4	3060	14.31
1	10000	20	5	1578	14.57
2	10000	01	1	352	Inf
2	10000	01	2	195	15.65
2	10000	01	3	205	15.65
2	10000	01	4	373	15.26
2	10000	01	5	291	Inf
2	10000	05	1	283	15.35
2	10000	05	2	529	15.18
2	10000	05	3	877	15.11
2	10000	05	4	355	15.18
2	10000	05	5	418	15.05
2	10000	10	1	1822	14.95
2	10000	10	2	628	14.88
2	10000	10	3	678	14.95
2	10000	10	4	959	14.75
2	10000	10	5	830	15.18
2	10000	15	1	1003	14.95
2	10000	15	2	2432	14.88
2	10000	15	3	1103	14.57
2	10000	15	4	999	14.45
2	10000	15	5	1221	14.40
2	10000	20	1	3474	14.75
2	10000	20	2	1247	14.65
2	10000	20	3	3469	14.57
2	10000	20	4	2495	14.31
2	10000	20	5	2176	14.57
1	15000	01	1	3051	15.95
1	15000	01	2	3004	15.48
1	15000	01	3	3199	Inf
1	15000	01	4	3611	15.48
1	15000	01	5	3516	15.35
1	15000	05	1	4173	15.18
1	15000	05	2	3642	14.81

C	P	R	T	Y	D
1	15000	05	3	3715	15.65
1	15000	05	4	4181	15.48
1	15000	05	5	3665	15.35
1	15000	10	1	4784	14.42
1	15000	10	2	4186	14.57
1	15000	10	3	4252	14.54
1	15000	10	4	4684	14.61
1	15000	10	5	3666	14.95
1	15000	15	1	5631	14.65
1	15000	15	2	4176	14.24
1	15000	15	3	4563	14.57
1	15000	15	4	4254	14.59
1	15000	15	5	3738	14.78
1	15000	20	1	4276	14.32
1	15000	20	2	3558	14.81
1	15000	20	3	4221	14.19
1	15000	20	4	3761	14.56
1	15000	20	5	3733	14.57
2	15000	01	1	538	15.95
2	15000	01	2	447	15.48
2	15000	01	3	452	Inf
2	15000	01	4	629	15.48
2	15000	01	5	443	15.35
2	15000	05	1	826	15.18
2	15000	05	2	1179	14.81
2	15000	05	3	1046	15.65
2	15000	05	4	1114	15.48
2	15000	05	5	1093	15.35
2	15000	10	1	5053	14.42
2	15000	10	2	2904	14.57
2	15000	10	3	3531	14.54
2	15000	10	4	2688	14.61
2	15000	10	5	2123	14.95
2	15000	15	1	8173	14.65
2	15000	15	2	3698	14.24
2	15000	15	3	6565	14.57
2	15000	15	4	5234	14.59

C	P	R	T	Y	D
2	15000	15	5	3636	14.78
2	15000	20	1	7051	14.32
2	15000	20	2	4008	14.81
2	15000	20	3	5887	14.19
2	15000	20	4	4845	14.56
2	15000	20	5	4936	14.57
1	20000	01	1	9761	15.26
1	20000	01	2	9498	15.95
1	20000	01	3	8739	15.18
1	20000	01	4	9922	15.65
1	20000	01	5	7227	15.95
1	20000	05	1	10304	15.18
1	20000	05	2	8441	14.65
1	20000	05	3	8376	15.05
1	20000	05	4	8631	15.05
1	20000	05	5	9649	14.75
1	20000	10	1	9618	14.61
1	20000	10	2	8546	14.57
1	20000	10	3	9786	14.37
1	20000	10	4	8634	14.61
1	20000	10	5	11688	14.65
1	20000	15	1	12470	14.49
1	20000	15	2	10996	14.15
1	20000	15	3	10200	14.19
1	20000	15	4	8765	14.05
1	20000	15	5	9677	14.24
1	20000	20	1	8370	14.31
1	20000	20	2	9648	13.96
1	20000	20	3	9793	14.27
1	20000	20	4	8710	14.30
1	20000	20	5	14261	14.46
2	20000	01	1	1587	15.26
2	20000	01	2	1609	15.95
2	20000	01	3	820	15.18
2	20000	01	4	1516	15.65
2	20000	01	5	818	15.95
2	20000	05	1	2273	15.18

C	P	R	T	Y	D
2	20000	05	2	2245	14.65
2	20000	05	3	2446	15.05
2	20000	05	4	3032	15.05
2	20000	05	5	2609	14.75
2	20000	10	1	9375	14.61
2	20000	10	2	4844	14.57
2	20000	10	3	5325	14.37
2	20000	10	4	5907	14.61
2	20000	10	5	7797	14.65
2	20000	15	1	15684	14.49
2	20000	15	2	9191	14.15
2	20000	15	3	8038	14.19
2	20000	15	4	8080	14.05
2	20000	15	5	8724	14.24
2	20000	20	1	10461	14.31
2	20000	20	2	12245	13.96
2	20000	20	3	11784	14.27
2	20000	20	4	11827	14.30
2	20000	20	5	21385	14.46
1	25000	01	1	4956	15.35
1	25000	01	2	3378	15.35
1	25000	01	3	2161	15.95
1	25000	01	4	3353	15.48
1	25000	01	5	7992	15.35
1	25000	05	1	16370	15.05
1	25000	05	2	23961	14.95
1	25000	05	3	24046	14.57
1	25000	05	4	20086	14.78
1	25000	05	5	18908	14.72
1	25000	10	1	16886	14.48
1	25000	10	2	18544	14.19
1	25000	10	3	17378	14.48
1	25000	10	4	18448	14.27
1	25000	10	5	19571	14.70
1	25000	15	1	19463	14.31
1	25000	15	2	20667	14.28
1	25000	15	3	20322	14.29

C	P	R	T	Y	D
1	25000	15	4	16338	14.18
1	25000	15	5	16918	14.05
1	25000	20	1	18952	14.54
1	25000	20	2	31391	14.16
1	25000	20	3	16597	13.95
1	25000	20	4	16482	14.40
1	25000	20	5	18713	14.45
2	25000	01	1	893	15.35
2	25000	01	2	439	15.35
2	25000	01	3	334	15.95
2	25000	01	4	448	15.48
2	25000	01	5	721	15.35
2	25000	05	1	4793	15.05
2	25000	05	2	9747	14.95
2	25000	05	3	9428	14.57
2	25000	05	4	11976	14.78
2	25000	05	5	4994	14.72
2	25000	10	1	12333	14.48
2	25000	10	2	10087	14.19
2	25000	10	3	11510	14.48
2	25000	10	4	11086	14.27
2	25000	10	5	11363	14.70
2	25000	15	1	27563	14.31
2	25000	15	2	33178	14.28
2	25000	15	3	27606	14.29
2	25000	15	4	16147	14.18
2	25000	15	5	17495	14.05
2	25000	20	1	23979	14.54
2	25000	20	2	27751	14.16
2	25000	20	3	24110	13.95
2	25000	20	4	23205	14.40
2	25000	20	5	23253	14.45
1	30000	01	1	9701	15.35
1	30000	01	2	24370	Inf
1	30000	01	3	13166	15.35
1	30000	01	4	3347	15.65
1	30000	01	5	3129	15.05

C	P	R	T	Y	D
1	30000	05	1	36528	14.75
1	30000	05	2	29630	15.18
1	30000	05	3	31776	14.61
1	30000	05	4	39331	14.84
1	30000	05	5	34135	14.57
1	30000	10	1	37479	14.18
1	30000	10	2	35608	14.21
1	30000	10	3	27151	14.33
1	30000	10	4	27950	14.11
1	30000	10	5	35853	14.40
1	30000	15	1	37747	14.65
1	30000	15	2	31978	13.93
1	30000	15	3	29341	14.22
1	30000	15	4	28225	14.26
1	30000	15	5	27734	14.20
1	30000	20	1	25424	14.37
1	30000	20	2	29836	14.37
1	30000	20	3	25018	14.37
1	30000	20	4	28686	14.01
1	30000	20	5	37765	13.98
2	30000	01	1	987	15.35
2	30000	01	2	2937	Inf
2	30000	01	3	1130	15.35
2	30000	01	4	579	15.65
2	30000	01	5	695	15.05
2	30000	05	1	13442	14.75
2	30000	05	2	9044	15.18
2	30000	05	3	9385	14.61
2	30000	05	4	10934	14.84
2	30000	05	5	23194	14.57
2	30000	10	1	31908	14.18
2	30000	10	2	19229	14.21
2	30000	10	3	31883	14.33
2	30000	10	4	20380	14.11
2	30000	10	5	20236	14.40
2	30000	15	1	40824	14.65
2	30000	15	2	30183	13.93

C	P	R	T	Y	D
2	30000	15	3	25589	14.22
2	30000	15	4	29755	14.26
2	30000	15	5	29616	14.20
2	30000	20	1	36198	14.37
2	30000	20	2	41532	14.37
2	30000	20	3	36532	14.37
2	30000	20	4	38329	14.01
2	30000	20	5	49392	13.98

Table S1: Extended data table for Experiment 1.

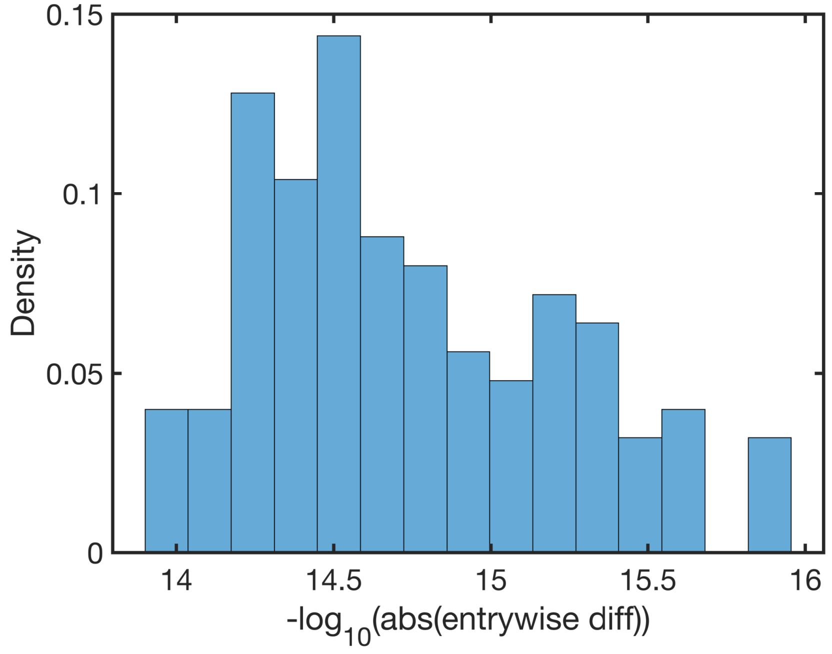

Figure S1: Histogram of $-\log_{10}$ values of the maximum absolute entrywise difference between the *kgen* and *naïve* algorithms. For each condition listed in Table S1 (in $-\log_{10}$ Cholesky space units), the maximum is taken over all phenotypes and all entries in the upper triangle of the Cholesky decompositions (leading to 125 items in the histogram). Values of ∞ (indicating equality in value of the methods up to numerpical precision) are suppressed.

Appendix C: The *kgen* software manual *v1*

The *kgen* software implements the methods described in the main text. A *Makefile* is provided (described in Appendix C.1), and command line options are provided (Appendix C.2), and the software is released under the open source BSD 2-clause license (listed in Appendix C.3).

Appendix C.1: Building the *kgen* software

The *kgen* software relies on the libraries *gsl* and *libqrupdate*. We provide a *Makefile* for the *kgen* software. This *Makefile* requires that headers and compiled libraries for *gsl* and *libqrupdate* be provided in the system's environmental path variables. A built copy of the *libqrupdate* software is provided with the *kgen* software, compiled on an Intel Xeon E5-2683 CPU using *gcc* 9.1.0.

The provided *Makefile* also makes use of Intel's *MKL* (Math Kernel Library). The build settings involved in *MKL* are coded for our system, but may be tuned to another system by using the forms provided at `https://software.intel.com/en-us/articles/intel-mkl-link-line-advisor`. Alternatively, through suitable modification of the *Makefile*, the *kgen* software can also be compiled against *netlib*'s *blas* and *lapack*.

TODO: refs.

Appendix C.2: Command line options

The following command line options are accepted by *v1* of the *kgen* software.

```
kgen --version

kgen [-v] [-t <thresh>] -m [tree|naive] <PFILE> <KFILE> <ODIR>

kgen [-d] <C1DIR> <C2DIR>

kgen -e
```

- `kgen --version`. Instructs *kgen* to print version information, and then exit.

- kgen [-v] [-t <thresh>] -m [tree|naive] ... Instructs *kgen* to create Cholesky decompositions for the kinship matrix <KFILE> and save the decompositions in <ODIR>/L00001.bin ... <ODIR>/L00XXXD.bin. Here d is the number of phenotypes provided in the file <PFILE>. The file <PFILE> must be a space sparated variable file for which the first line is a space separated header line with one string per phenotype (separated by spaces). Each line of the file <PFILE> indicates the phenotype records for a sample, and there must be n lines following the header (n is the number of samples). Each record of each line must contained the observed phenotype value (or the string NAN in case that record is missing). The file <KFILE> must contain a space separated file (with no header) in which entry i_1, i_2 contains the genetic similarity between subjects i_1 and i_2 (with $1 \leq i_1 \leq n$ and $1 \leq i_2 \leq n$). The files <PFILE> and <KFILE> must not have sample ID rows or columns (instead, samples must be presented in the same order in both files).

  The *kgen* software creates the directory <ODIR> and then uses either the *kgen* method (if the option -m tree is provided) or the naïve (if the option -m naive is provided) to create the d Cholesky decompositions. The decompositions are stored in a sparse format as serialised 64-bit doubles (according to the local encoding). The first n doubles are the first row of the Cholesky decomposition, and the next $n-1$ doubles are the second row (with the 0s in the lower triangle removed) and so on. If the flag v (verbose) is provided, then additional information about the progress and function of the methods are provided. To preserve numerical stability, we recommend that a small number ε be added to the diagonal of the matrix in <KFILE>. That number can be modulated by providing -t <thresh>, here <thresh> is the positive real number ε. The default value of ε is 10^{-3}.

  The files <KFILE> and <PFILE> can also be provided in a compact form (instead of as space separated variable files) as n^2 serialized doubles (for <KFILE>) and nd serialized 64-bit doubles (for <PFILE>) in column-major form. Here missing data in <PFILE> must be indicated using a 64-bit representation of the symbol NAN. This compact form is read if the filename ends in the string .bin. In this case, the values of n and d are inferred from the file sizes.

- kgen [-d] <C1DIR> <C2DIR>. Instructs *kgen* to find the maximum

absolute entrywise difference between the Cholesky decompositions in `<C1DIR>` and those in `C2DIR`. (This is used in Experiment 1 in the main text to report any discrepency between the solutions found by the two methods.)

- `kgen -e`. Instructs *kgen* to print the machine epislon, and then exit.

Appendix C.3: License for the *kgen* software


```
kgen v1. Copyright (c) 2019. Lloyd T. Elliott.

Redistribution and use in source and binary forms, with or
without modification, are permitted provided that the
following conditions are met:

1. Redistributions of source code must retain the above
copyright notice, this  list of conditions and the
following disclaimer.

2. Redistributions in binary form must reproduce the above
copyright notice, this list of conditions and the following
disclaimer in the documentation and/or other materials
provided with the distribution.

THIS SOFTWARE IS PROVIDED BY THE COPYRIGHT HOLDERS AND
CONTRIBUTORS "AS IS" AND ANY EXPRESS OR IMPLIED WARRANTIES,
INCLUDING, BUT NOT LIMITED TO, THE IMPLIED WARRANTIES OF
MERCHANTABILITY AND FITNESS FOR A PARTICULAR PURPOSE ARE
DISCLAIMED. IN NO EVENT SHALL THE COPYRIGHT HOLDER OR
CONTRIBUTORS BE LIABLE FOR ANY DIRECT, INDIRECT,
INCIDENTAL, SPECIAL, EXEMPLARY, OR CONSEQUENTIAL DAMAGES
(INCLUDING, BUT NOT LIMITED TO, PROCUREMENT OF SUBSTITUTE
GOODS OR SERVICES; LOSS OF USE, DATA, OR PROFITS; OR
BUSINESS INTERRUPTION) HOWEVER CAUSED AND ON ANY THEORY OF
LIABILITY, WHETHER IN CONTRACT, STRICT LIABILITY, OR TORT
(INCLUDING NEGLIGENCE OR OTHERWISE) ARISING IN ANY WAY OUT
OF THE USE   OF THIS SOFTWARE, EVEN IF ADVISED OF THE
POSSIBILITY OF SUCH DAMAGE.
```

References

Benoıt, E. (1924). Note sur une méthode de résolution des équations normales provenant de l'application de la méthode des moindres carrésa un systeme d'équations linéaires en nombre inférieura celui des inconnues. (Procédé du Commandant Cholesky). *Bulletin Géodésique*, *2*(1).

Hájek, J. (2012). *libqrupdate.* `https://sourceforge.net/projects/qrupdate/`. (Accessed: Summer 2019)

Osborne, M. A., Rogers, A., Roberts, S. J., Ramchurn, S. D., and Jennings, N. (2010). Bayesian Gaussian process models for multi-sensor time-serie prediction. In *Inference and learning in dynamic models.* Cambridge University Press.
